# The Impact of Stopping Medications and Introducing a Whole Food Plant-Based Diet on Patients Living with Multiple Sclerosis – A Report of Two Cases

**DOI:** 10.1177/15598276221141403

**Published:** 2022-12-01

**Authors:** Monty Cuthbert, Marta Lewandowska, Laura Freeman, Conor Devine, Karen Lee, Shireen Kassam

**Affiliations:** 156800 Royal Sussex County Hospital, Brighton, UK (MC, ML); Plant Based Health Online, Bordon, UK(LF, SK); Conor Devine, Belfast, UK (CD); The Sensitive Foodie, Brighton, UK (KL); 4616 King’s College London, London, UK (SK); and University of Winchester, Hampshire, UK (SK)

**Keywords:** multiple sclerosis, lifestyle, whole food plant-based diet, disease-modifying therapy

## Abstract

More than 2 million people live with multiple sclerosis worldwide and the prevalence has been increasing over time. Patients living with multiple sclerosis often explore diet and lifestyle interventions as a means of managing their symptoms and reducing reliance on medication; yet, these approaches are rarely discussed with their physicians. Currently, there is a lack of evidence on when to stop disease-modifying therapies (DMT), and recent research showed no statistically significant difference in the time between relapses when comparing participants who stopped DMT to those who did not, especially over the age of 45. This case report presents 2 patients with multiple sclerosis who made an informed decision to stop their DMT medications and have been managing their condition with a whole food plant-based diet and a healthy lifestyle approach. Over the period of 5 to 6 years since stopping the medications, each patient only had 1 multiple sclerosis flare-up to date. In the report, the focus is on the impact of diet on multiple sclerosis. It adds to currently available literature and encourages further research in the field of managing multiple sclerosis with lifestyle interventions.

‘Saturated fat consumption from animal-derived foods has consistently been associated with an increased incidence of MS’.

## Introduction

Multiple sclerosis (MS) is a demyelinating disease of the central nervous system. The main symptoms that patients present with include fatigue, optic neuritis, paraesthesia and loss of balance.^1^ The incidence is higher in Western countries and it has been suggested that this may be in part related to diet and lifestyle factors. In the United Kingdom (UK), approximately 6700 people are diagnosed with MS each year.^2^ In 2016, it was estimated that more than 2.2 million people lived with MS worldwide – an increase of approximately 10% from 1990.^2^ On average, a person with MS has a 33 to 100% chance of relapse per year (or experiences 1 relapse every 2 years).^3^ This is variable, however, and the frequency of relapses decreases with time since diagnosis as well as with age.^4^ Disease modifying therapies (DMTs) targeting the immune system are the mainstay of treatment and in general patients rarely manage to eliminate medications.^1^

Patients living with MS often explore diet and lifestyle interventions as a means of managing their symptoms and reducing reliance on medication, yet these approaches are rarely discussed with their physicians.^5^ In general, healthy dietary patterns centred around whole plant foods, whilst minimising the consumption of animal-derived and processed foods are associated with lower levels of inflammation and improvement in symptom burden and quality of life.^6^ There are 2 broad dietary approaches promoted in the public domain. A low saturated fat, plant-predominant approach pioneered by Dr Roy Swank and a modified Paleolithic diet pioneered by physician and patient Dr Terry Wahls.^7^ A plant-based approach is more attractive given the well documented benefits for prevention of cardiovascular disease, type 2 diabetes, inflammatory bowel disease, anxiety and depression, all conditions that are more prevalent in people living with MS.^8^

This case report presents 2 patients with MS who were able to stop their DMT medications and have been managing their condition with a whole food plant-based diet (WFPBD) and a healthy lifestyle approach.

## Methods

Two patients were asked to describe their experience of living with MS. Information was gathered via email (asking for a personal account of their experience), clinic letters, blood tests results, imaging and telephone discussions. The patients were asked to fill in a table outlining their dietary habits before and after adapting a WFPBD (Table 1).

**Table 1.** Dietary Pattern Before and After Adopting Whole Food Plant-Based Diet (WFPBD).

	Diet Before WFPBD	Diet After WFPBD
Patient A		
Grains	Rice, brown rice, oats	Rice, brown rice, oats
Pulses	Baked beans	Baked beans, lentils (all kinds), chickpeas, peas, black eyed peas, kidney beans
Vegetables	Carrots, green leafy vegetables, broccoli	Peppers, carrots, cauliflower, broccoli, kale, onions (big portions with every meal)
Fruit	Apples, bananas	Bananas, oranges, apple, pears, melon, blueberries
Nuts and seeds		Almond nuts, cashew nuts
Processed foods	Bread, sausage rolls, chocolate, cakes, biscuits, cheese, pizza, butter	Occasional sweets
Animal products	Chicken, beef, fish	
Patient B		
Grains	White pasta, white rice, wholemeal flour, oats, couscous	Wholemeal flour including spelt, emmer flour, wholegrain red and black rice, millet, buckwheat, quinoa, oats, barley
Pulses	Red and green lentils, chickpeas, kidney beans, haricot beans	Red, green and black lentils, white beans (butter, cannellini), black beans, flageolet, haricot, split yellow peas, fava beans, soya beans (fresh and dried), organic tofu
Vegetables	Potatoes, broccoli, cabbage, peas, salad, peppers, spinach, tinned tomatoes (a lot) and tinned sweetcorn, onion, garlic, cabbage. Total vegetable consumption 3–4 portions/day.	Potatoes, sweet potatoes, all root vegetables, leafy greens, salad greens, radish, tomatoes, cucumber, sweet corn, green beans, peas, broccoli, peppers, avocado, cabbages, squash, celery, mushrooms, onions and leeks, spices/herbs – turmeric, ginger, garlic, coriander, parsley. All seasonal, ideally organic. Total vegetable consumption 8–10 portions/day.
Fruit	Apple, banana, seasonal berries. Total fruit consumption 1–2 portions/day.	Apple, pear, banana, berries (fresh and frozen), passion fruit, plum, peach, melon, mango (when in season), grapefruit, lemon and lime. Total fruit consumption 2–3 portions/day
Nuts and seeds	Coconut – dried and fresh, coconut milk, peanuts, cashew, almond, walnut, sunflower, pumpkin. Total nuts and seed consumption 2 portions/day.	Flaxseed daily plus either chia seeds, pumpkin, hemp or sesame seeds. Total seeds consumption 2 portions/day. Walnuts, cashew, almonds, hazelnut, pecans, pine nuts. Total nuts consumption 1 portion/day.
Processed foods	Coconut milk and oil, oil, baked beans, vegan spread, vegan sausages and burgers, chips, fried food (when eating out), oat cakes, vegan ice cream, sauces, take away and eating out twice a week.	Dairy-free milk (2 servings/day). Tamari, maple syrup, Dijon mustard, cider vinegar, nutritional yeast (not daily, used in cooking as needed). Booja booja vegan ice cream (no coconut or palm oil), Alpro custard, Linda McCartney vegan sausage (no palm oil) (all rare treats).
Animal products		

## Case Presentation

### Patient A

Patient A is a 44-year-old male living in the UK who was diagnosed with MS in 2007, aged 29. His first flare-up occurred while on holiday abroad. He experienced left-sided limb weakness in August 2006. Upon return to the UK, a brain and spine MRI showed ‘lesions in the upper cervical cord/lower brainstem consistent with inflammatory demyelination’. No medications were commenced at the time.

Six months later, he reported persistent ‘electrical buzzing in the left side of his chest’ and lamotrigine tablets were introduced. He was complaining of ongoing globus pharyngeus.

In August 2007, Patient A reported lightheadedness, nausea, right upper limb weakness and tingling in the fingers. An MRI showed supratentorial demyelination. A diagnosis of relapsing remitting MS was made and the patient was commenced on a reducing course of oral dexamethasone. As symptoms of malaise persisted, interferon beta-1a was introduced in October 2007.

Interferon beta-1a caused constant flu-like symptoms, particularly in the morning. In February 2008, the treatment was changed to glatiramer acetate. The patient adhered to the medication well and reported good disease control. He continued regular gym workouts including strength and aerobic training.

Patient A remained on glatiramer acetate treatment until March 2016 with 1 major relapse in 2010. He ran a few half-marathons and a full marathon. However, he noticed waning effects of the medication in 2015, when he was experiencing flu-like symptoms for 7 weeks, along with paraesthesia and neuralgia.

In April 2016, Patient A decided to stop glatiramer acetate completely and commence a WFPBD (Table 1). The decision was based on his own research and not discussed with the clinical practitioner. At the time, his VIAASDC (Vienna Innsbruck DMT discontinuation score based on age, activity on MRI and duration in stable course) was 4, meaning a 90% likelihood of disease reactivation in 5 years.^9^

Since starting a WFPBD, he has only experienced 1 MS flare-up. It presented in April 2017 as right-sided limb paraesthesia and monoparesis. A repeated MRI in September 2018 showed no lesions specific to inflammatory demyelination, and a few lesions in the peritrigonal region which could represent previous areas of inflammatory demyelination. Patient A does not have any regular follow-up appointments relating to MS.

He has been running his own business and remained physically active to date, including completing Iron-Man competitions and several triathlons.

### Patient B

Patient B is a 53-year-old female who was diagnosed with MS at the age of 47, in 2016. Her first symptoms occurred in May 2015, that of right-sided optic neuritis. She experienced another episode of optic neuritis in the left eye in October 2015 followed by left-sided arm neuralgia and weakness. MRI brain in December 2015 showed at least 2 non-enhancing subcortical lesions. Finally, a lumbar puncture in March 2016 showed evidence of oligoclonal bands in the cerebrospinal fluid and Patient B was officially diagnosed with relapsing remitting MS.

Soon after the MS diagnosis, Patient B read a book called ‘Overcoming Multiple Sclerosis: The Evidence-Based 7 Step Recovery Program’ by Dr George Jelinek.^10^ She had already been following a vegan lifestyle; however, after reading the book, she decided to commence a WFPBD, and supplement with further vitamin D and cold-pressed flaxseed oil (Table 1).

Patient B commenced treatment with DMTs in September 2016, starting with dimethyl fumarate. This caused a number of side-effects including flu-like symptoms, dysgeusia, phantosmia and deranged liver function tests. On discussion with the MS Specialist Nurse, a joint decision was made to stop taking the medication in December 2016.

Patient B continued experiencing left arm neuralgia and right leg paraesthesia; therefore, she agreed to commence glatiramer acetate treatment in September 2017. After 6 weeks of treatment, wheals started appearing at injection sites and therefore, the medication was stopped in November 2017. Furthermore, a stage I melanoma was resected in December 2017. Since then, no further MS DMTs have been commenced, as per the patient’s preference and in agreement with the clinical practitioner. At the time, her VIAASDC was 3, giving her a 40% chance of disease recurrence in 5 years.^9^ She has only had 1 flare-up of MS since stopping medications, in November 2019 – right optic neuritis. She remains under the care of a consultant neurologist and has annual follow-up appointments.

She has remained physically active with regular yoga sessions and half-marathon running.

## Outcome

**Figure 1.**
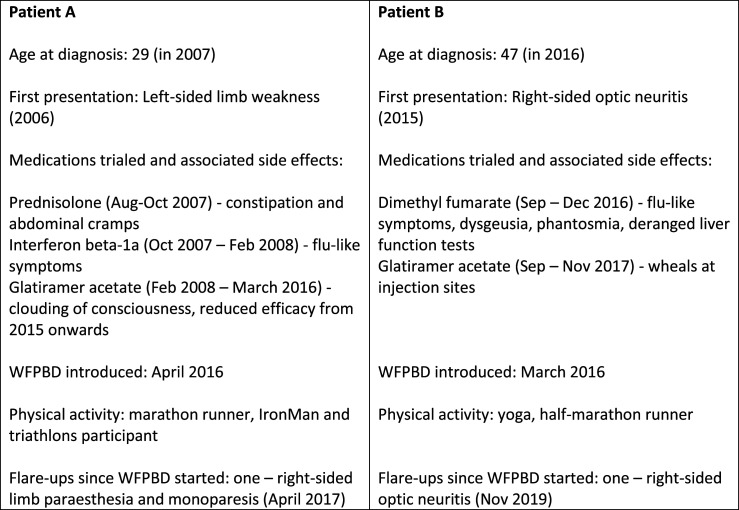
Summary of participants’ profiles.

These are case studies describing 2 people with MS (pwMS) who experienced considerable DMTs side effects and therefore decided to stop the DMTs and manage their disease with a WFPBD instead. Their decision was based on their own research, and Patient B involved the clinical team in her decision-making process. Their food habit changes are summarised in Table 1, and patient summary along with the DMTs side effect profile is depicted in Figure 1. At last follow-up in September 2022, 6 years and 6 months after Patient A discontinued DMT and commenced a WFBPD, and 5 years and 10 months after Patient B discontinued DMT, they had both experienced 1 episode of disease reactivation since the change, which, over the course of 5 years, was 40% likely in case of Patient A, and 90% likely in case of Patient B as per the VIAASDC.^9^

## Discussion

We have presented 2 patients who have successfully managed MS using a WFPBD, whilst eliminating the use of medication. Whilst this approach is not going to be suitable for all pwMS, it highlights what may be possible for some patients who make intensive lifestyle changes. There are a number of plausible reasons for the success of such an approach. These include the anti-inflammatory and immunomodulatory properties of a plant-based diet^11^ and the positive impact on the gut microbiome.^12^ Guidelines on discontinuing DMTs in MS are limited and it mainly occurs when side effects of treatment negatively impact quality of life. A comprehensive review from Knox and colleagues concluded that there is a lack of evidence on when to stop DMTs and in the decision-making process, ‘shared decision making and consideration of ethical complexities’ should take place.^13^ A recent longitudinal study showed no statistically significant difference in the time between relapses when comparing participants who stopped DMT to those who did not.^14^ In particular, patients over the age of 45 were more likely to have a stable disease course, while those under 45 were more likely to relapse when stopping the medications.^14,15^ In another study, participants who stopped administering injectable DMT after a long relapse-free period of time had a similar frequency of relapse as those continuing medications, but an increased risk of disability progression.^16^

### MS and Fruit and Vegetable Intake

The positive impact of increasing fruit and vegetable consumption is a consistent finding in pwMS. A recently published study showed that healthy diet patterns with higher consumption of fibre, fruit and vegetables were associated with better mental and physical health and well-being.^17^ The positive influence of consuming fruit and vegetables was noted in the large NARCOMS trial where a high fruit and vegetable intake was associated with a decreased disability score.^18^ Frequently compared Wahls and Swank dietary interventions for relapsing-remitting MS, although markedly different, both recommend a higher than average intake of fruit and vegetables – at least 4 cups of fruit and vegetables a day in the Swank diet and 6 cups in the Wahls diet.^7^ Reportedly, both of these lead to reduction in fatigue and increase in quality of life in between 50% to 75% of study participants; in the studies, those effects were maintained for 24 weeks – the duration of follow-up.^19^ In addition, increased intake of vegetables may help prevent further relapses. In a study of paediatric pwMS, ‘each additional cup of vegetables decreased the hazard of relapse by 50%’.^20^

### MS and Animal Foods

Saturated fat consumption from animal-derived foods has consistently been associated with an increased incidence of MS. Although the relationship may not be causal, there is evidence supporting the reduction of saturated fat by reducing or eliminating the consumption of meat and dairy. In a longitudinal study from the UK, elimination of meat consumption was associated with reduced progression of disability although dairy consumption showed mixed results.^21^ In another UK-based study, there was a weak positive correlation between red meat intake and anxiety, depression and pain.^4^ Meat and dairy consumption were positively associated with an increased depression score in an Australian cohort.^22^ Although more research would be beneficial, given the remarkable long-term results of the low saturated fat Swank protocol^17^ and the beneficial effects on inflammation^23,24^ and the gut microbiome,^25^ replacing saturated fat with healthy plant sources of fat is a sensible approach. In addition, this type of dietary approach has significant benefits for cardiovascular and metabolic health.^26^

Despite this, evidence suggests that pwMS frequently commence fish oil supplementation after their diagnosis.^27^ Patient B, as a vegan, started using flax seed oil to supplement her diet after learning that she had MS. It is richer in alpha-linolenic acid than fish and algae oils, which have a higher proportion of the other poly-unsaturated fatty acids (PUFAs) known to be beneficial in MS, such as eicosapentaenoic acid and docosahexaenoic acid. Although flaxseed oil is considered inferior to fish and algae oils, there is scope for further research into the benefits of all PUFAs and short-chain fatty acids in pwMS.^28,29^

### The Impact of Diet on MRI Results

Few data are available on the impact of diet on disease activity as observed on brain MRI. Perhaps this is because quality of life, fatigue and the number of relapses carry a larger clinical significance and are therefore preferred objectives in study designs. In addition, improvements in MRI findings may take longer than those experienced by the patient.

Yadav and colleagues published a randomised controlled trial on the effect of a very low-fat plant-based diet on brain MRI and other clinical outcomes.^30^ The results failed to show significant improvement in brain MRI outcomes over 1 year, although patients experienced less fatigue, had reductions in body weight and improvements in metabolic markers.

### MS and Gut Microbiome

Gut dysbiosis is now considered integral to the pathogenesis of MS.^31^ PwMS have reduced microbiome diversity during flare-ups compared to those in remission; for example, there is a reduced number of bacteria which have active anti-inflammatory properties.^12^ A more recent study also showed that patients with more disability had a greater abundance of bacteria species typical for other autoimmune and inflammatory diseases.^32^ Furthermore, they showed a correlation between high intake of meat and dairy and raised Th17 level – cells that promote inflammation in MS.^32^

Overall, strict vegan diets rich in fibre have been shown to contribute to maintaining a more diverse and healthier gut flora that, among other benefits, exhibits anti-inflammatory properties.^11,33^

### Dietary Guidelines in MS

Currently, NICE (National Institute for Health and Care Excellence), the main body creating evidence-based clinical guidelines for healthcare professionals in England, only includes advice on smoking, vaccinations, pregnancy and exercise in their ‘Modifiable risk factors for relapse or progression of MS’.^34^ A Cochrane review also found insufficient evidence to support any dietary intervention in managing MS symptoms.^35^ This is reflective of the limited availability of evidence supporting particularly dietary choices as well as the lack of large randomised dietary intervention trials. However, it is increasingly difficult to ignore the consistent epidemiological data and lived experience of pwMS that report substantial benefits of a high fibre, plant-predominant diet pattern.

## Learning Points

1. Little is known on how MS patients perform without DMT treatment.
2. An increased intake of fruit and vegetables may reduce the number, rate and severity of further MS relapses as well as reduce fatigue and increase self-reported quality of life.
3. Lowering saturated fat intake through the reduction or elimination of animal foods may improve both physical and psychological well-being.
4. More research is needed in the area of lifestyle intervention on MS symptoms.
